# Megachiropteran bats profoundly unique from microchiropterans in climbing and walking locomotion: Evolutionary implications

**DOI:** 10.1371/journal.pone.0185634

**Published:** 2017-09-28

**Authors:** Rick A. Adams, Richard T. Carter

**Affiliations:** 1 School of Biological Sciences, University of Northern Colorado, Greeley, Colorado, United States of America; 2 Department of Biology, University of Dayton, Dayton, Ohio, United States of America; Texas A&M University College Station, UNITED STATES

## Abstract

Understandably, most locomotor analyses of bats have focused on flight mechanics and behaviors. However, we investigated nonflight locomotion in an effort to glean deeper insights into the evolutionary history of bats. We used high-speed video (300 Hz) to film and compare walking and climbing mechanics and kinematics between several species of the suborders Megachiroptera (Pteropodidae) versus Microchiroptera (Vespertilionidae and Phyllostomatidae). We found fundamentally distinctive behaviors, functional abilities, and performance outcomes between groups, but nearly homogeneous outcomes within groups. Megachiropterans exhibited climbing techniques and skills not found in microchiropterans and which aligned with other fully arboreal mammals. Megachiropterans climbed readily when placed in a head-up posture on a vertical surface, showed significantly greater ability than microchiropterans to abduct and extend the reach of their limbs, and climbed at a greater pace by using a more aggressive ipsilateral gait, at times being supported by only a single contact point. In addition, megachiropterans showed little ability to employ basic walking mechanics when placed on the ground, also a pattern observed in some highly adapted arboreal mammals. Conversely, microchiropterans resisted climbing vertical surfaces in a head-up posture, showed significantly less extension of their limbs, and employed a less-aggressive, slower contralateral gait with three points of contact. When walking, microchiropterans used the same gait they did when climbing which is representative of basic tetrapod terrestrial mechanics. Curiously, megachiropterans cycled their limbs significantly faster when climbing than when attempting to walk, whereas microchiropterans cycled their limbs at significantly faster rates when walking than when climbing. We contend that nonflight locomotion mechanics give a deep evolutionary view into the ancestral es locomotor platform on which flight was built in each of these groups.

## Introduction

The evolution of flight in mammals has resulted in one of the most diverse and ecologically prolific orders, the Chiroptera. Aspects of bat flight from both mechanical and ecological perspectives have dominated the literature [1]. Although these topics are of obvious importance, studies of nonflight locomotion may give novel insights into the evolution of this group. Specifically, comparing the mechanics and kinematics of walking and climbing may reveal deep evolutionary history evident in the ancestor of bats before the origin of flight.

The origin of bats and the evolutionary relationship between the suborders Megachiroptera (Family: Pteropodidae) and Microchiroptera (all other families of bats) is contentious and unresolved (A comprehensive discussion of these perspectives can be found in [1]). Some argue that the profound morphological differences between these groups evidences a diphyletic origin for bats, whereas others contend that bats show clear genetic monophyly. Despite the different perspectives, agreement can be reached that selection for aerodynamics and the physics of flight have conformed the morphology of all bats to certain expectations. However, other forms of locomotion used by bats may not have been caught up in the selective filter for flight and the foundational locomotor platform on which flight was originally built would likely be maintained on some level in all descendants.

Another contentious issue concerns if the direct ancestor of bats was arboreal or terrestrial. Some hypothesize that bats must have been derived from an arboreal ancestor, proceeded through a gliding stage into flight (trees-down hypothesis) [1]. Others argue that no gliding stage occurred and that mammals went directly to flapping locomotion, which opens up the possibility of a ground-up origin for flight [2, 3]. Accordingly, analysis of walking and climbing may help answer these questions because the intrinsic locomotor platform (terrestrial or fully arboreal) on which flight was built should help elucidate the origin. Indeed, if bats derived from a fully arboreal ancestor, consistent evidence of adaptive arboreal mechanics and kinematics would more likely than not be retained in descendants as would terrestrial mechanics and kinematics if that was the platform for flight.

Limited descriptions of terrestrial locomotion do exist for a few species of microchiropteran bats. Van Dan Brink [4], in his *A Field Guide to the Mammals of Britain and Europe* illustrated basic walking/running tracks of the noctule (*Nyctalus noctula*). Lawrence [5] described the walking and running gaits of three microchiropteran bat species from the Family Vespertilionidae, *N*. *noctula*, the serotine (*Eptesicus serotinus*) and the common pipistrelle (*Pipistrellus pipistrellus*), by having them walk over fine-grain soil covered in talc powder. He found that these bats used a typical mammalian gait with fore- and hindlimbs moving in diagonally opposite pairs [i.e. contralateral gait, right front limb (RF), then left hindlimb (LH), followed by left front limb (LF), then right hindlimb (RH)].

Lawrence [5] further observed what he termed "running" in *N*. *noctula* and *P*. *pipistrellus* where the forearms were more splayed than in walking and individuals moved with greater speed while elevating the tail and chin, indicating greater overall balance. In both species, the footfall pattern in running was the same as in walking, but the extent of forward motion of both the fore- and the hindlimbs were more restricted when running. He also found that *N*. *noctula* were capable of leapfrogging for which the footfall pattern was LF, RF, LH, RH.

Dietz [6] designed a set of walking experiments using five vespertilionid bats (reported as *Myotis velifer*, *M*. *yumanensis*, *Antrozous*, *Eptesicus*, and *Pipistrellus*) and one species from the family Molossidae noted only as *Tadarida*. He found that whereas vespertilionid bats carried their bodies at various angles to the substrate when walking, the molossid, *Tadarida*, held their bodies parallel to the substrate, which may be more efficient. Dietz [6] found that the bats he tested were fundamentally well-coordinated walkers and, of the species observed, *Antrozous* was the most versatile and used a wide variety of gaits, speeds, and body positions. In addition, even though some species of bats appear to have lost the motivation, or ability, for walking as adults (*Leptonycteris* and *Macrotis*), some efforts at terrestrial locomotion were observed in youngsters between postpartum days 1 to 25 [6].

Of course, some extant bat species have evolved remarkably good walking and sprinting skills, morphologies, and behaviors [7]. These include the vampire bats (*Desmodus rotundus* and *Diaemus youngi*, Phyllostomidae), the ground-foraging pallid bat, (*Antrozous pallidus*, Vespertilionidae) as well as two species from the family Molossidae (*Cheiromeles torquatus* and *C*. *parvidens*). An endemic species to New Zealand, the lesser short-tailed bat (*Mystacina tuberculate*, Mystacinindae) also forages terrestrially and has excellent walking and scampering ability [7, 8]. The energetics of walking or sprinting in bats is not well understood, with the exception of a molossid (*Molossus currentium*). Voigt et al. [9] quantified energetic cost of sprinting in *M*. *currentium* as compared to six rodent species and found that peak metabolic rates were significantly higher in *M*. *currentium* than in the rodents tested. In addition, the cost of transport (j kg^-1^m^-1^) was 10 times higher for *M*. *currentium* when scampering than it was when flying. Virtually no studies have quantified walking mechanics in megachiropterans and likewise no published research on climbing mechanics in bats appears to exist.

Although little is understood about walking and climbing, bats use these forms of locomotion to access and leave roosting sites, move around on the wall of caves, as well as the trunks and limbs of trees to access food [1]. Undeniably nonflight locomotion is involved in the daily/nightly lives of bats and undoubtedly is a significant factor in survival. In this paper we compared climbing and walking locomotion in several species of flying foxes (Megachiroptera: Pteropodidae) with that of several species of Microchiroptera (Vespertilionidae and Phyllostomidae). The working hypothesis for this study was that bats are a monophyletic group derived from arboreal ancestry.

## Materials and methods

### Sites

We visited two captive facilities [Lubee Bat Conservancy (LBC), Florida, an outdoor facility and Organization for Bat Conservation (OBC) in Michigan, an indoor facility] and filmed a variety of available bat species, which provided a range of body sizes and phylogenies. The megachiropterans available for filming were *Eidolon helvum*, *Rousettus aegyptiacus*, *Pteropus hypomelanus*, *P*. *pumilus* and *Cynopterus brachyotis*. The microchiropterans available for filming were the vespertilionids *Eptesicus fuscus* and *Nyticeius humeralis* and the phyllostomids *Artibeus jamaicensis* and *Carollia perspicillata*. One of us (RAA) also filmed climbing and walking in two fringed myotis (*Myotis thysanodes*, Vespertilionidae) that were caught in a mist net in a flyway in Geer Canyon (T 13, 473336.09 m E, 44444133.23 m N), Boulder, Colorado and released immediately after filming.

Climbing individuals were filmed using the caging that they were accustomed to, or in the case of the wild-caught *M*. *thysanodes*, a 12.7 x 12.7mm hard plastic mesh that matched the caging at OBC allowing for comparisons of climbing performance between captive and wild individuals. It should be noted that the caging at LBC was double-wired which at times caused claws to get momentarily stuck between wires. Although this did not alter limb mechanics or motions, it did slow down climbing rates somewhat; therefore performance comparisons were done only on individuals filmed climbing the same mesh used at OBC. Walking was filmed from individuals placed either a table or platform covered with thin padding (Microchiroptera) or individuals that were placed on the ground (Megachiroptera).

### Filming

Two Casio Exilim high-speed cameras (Casio Computer Co., LTD, Tokyo, Japan) set at 300 frames per second with a resolution of 320 x 240 pixels were used to film bats. Because large-bodied megachiropterans had to be placed on the ground to walk, we filmed them using a single camera from a dorsal position. All other filming used two cameras set off simultaneously, one positioned lateral to the bat and the other positioned in dorsal aspect. All cameras were placed on tripods and stationary.

### Positioning bats for climbing and walking trials

Individual bats were handled by each organization's skilled caretakers, or in the case of the wild-caught bats, by RAA. Individuals were filmed 1–3 times each. For climbing trials, all individuals were placed head-up onto a screen by the handler and released once the bats were clearly secured to the screen. For walking trials, microchiropterans were released from hand onto a table platform, whereas megachiropterans were placed on the ground and released while filmed from the dorsal aspect only. If an individual did not climb or walk immediately, it was prompted by gently touching the tail region with the handler's index finger. In some cases it took 2–3 trials for individuals to climb or walk in a manner (distance and within camera frames) that allowed for analysis of mechanics. If an individual immediately climbed or walked through two or more limb cycles within the filming frame of the cameras, we did not refilm that individual. If an individual refused to perform or showed stress (struggled to escape), s/he was retired from the study. All methods of data collection were approved by the University of Northern Colorado IACUC (protocol # 1510C-RA-B-18) and the Lubbe Bat Conservance IACUC (protocol# 16CR_01).

### Analysis

Although several video clips of individuals may have been taken, we chose a single clip for which the individual climbed or walked the longest distance within the camera frames to examine and evaluate. Video clips were analyzed using MaxTrax Motion Analysis Software (Innovations Systems, Inc., Columbiaville, MI, USA). Initiation of a climb or walk was defined as beginning movement of a limb or limbs. Landmarks were chosen according to specific kinematics to be measured and placed manually at designated points (landmarks shown in Results section) and angles between landmarks were determined using the software's angle calculation tool. Means for the left and right limb measurements of comparable angle measurements for each individual were compiled. These means for the left and right limb angles were then run in a single Student's T-Test comparing those means across all samples taken within megachiropterans and within microchiropterans. If no significant differences were found between the means of individuals within megachiropterans and within microchiropterans, angles measured for left and right limbs of each group were combined and compared to each other using a Student's T-test. Similarly, one-way ANOVA was used for comparisons of multiple species within each group to test for significant differences among species (microchiropterans and megachiropterans).

Distances bats travelled when climbing were calculated by using known mesh dimensions and head lengths measured (mm) using the ruler tool in MaxTrax Motion Analysis Software. Limb cycle sequences were defined as initiated movement by the first of the four limbs for either climbing or walking and each sequence ended by initiation movement of that same limb after extension of the other three limbs. Time stamps provided by MaxTrax Motion Analysis Software were used to determine timing of sequences and these data were compared between megachiropteran and microchiropteran bats using a Mann Whitney U test. We built gait diagrams constructed using the methodology of Hildebrand [10] to compare initiation patterns of walking and climbing.

## Results

### Behaviors associated with vertical climbing

We filmed individuals from five species of microchiropteran bats (*Eptesicus fuscus*, N = 2; *Myotis thysanodes* (N = 2), *Nycticeius humeralis*, nonvolant (i.e. incapable of flight), N = 1; *Artibeus jamaicensis*, N = 1; and *Carollia perspicillata*, N = 1) all of which were captive individuals with the exception of two *M*. *thysanodes*. We could get *C*. *perspicillata* to climb a few paces, but individuals would not walk, instead they immediately launched into flight. For the other microchiropteran species, when individuals were placed head-up on a vertical screen, they would walk or climb a few paces, but the overall tendency was to reorient into a head-down position and either to crawl up back-wards or, more commonly, to immediately take flight. Big brown bats (*E*. *fuscus*) and fringed myotis (*M*. *thysanodes*) climbed readily through several limb cycles. The only microchiropteran that did not attempt to reorient its body to a heads-down position was *N*. *humeralis* that was a nonvolant individual for which climbing and walking were the only available modes of locomotion. This individual came to OBC from another facility and thus the origin of his injuries was unknown. Although, this individual could not fly, walking and climbing mechanics showed no obvious divergence from other microchiropterans. Indeed, like other microchiropterans, this individual climbed at an angle as opposed to a direct vertical ascent with its body held tightly to the vertical surface.

Megachiropterans tested were (*Pteropus hypomelanus* (N = 6), *P*. *pumilus* (N = 3), *Rousettus aegyptiacus* (N = 2), *Eidolon helvum*, (N = 3), and *Cynopterus brachyotis* (N = 2). For these species, the immediate tendency was to climb vertically head-first up the screen to the ceiling and then onto the ceiling and hang head-down. The body was commonly held less tightly to the screen compared to microchiropterans to allow for the hindlimbs to be moved beneath and ahead of the forelimb. This was not quantified in any formal way.

### Angle of the elbow during vertical climbing

**Fig 1:**
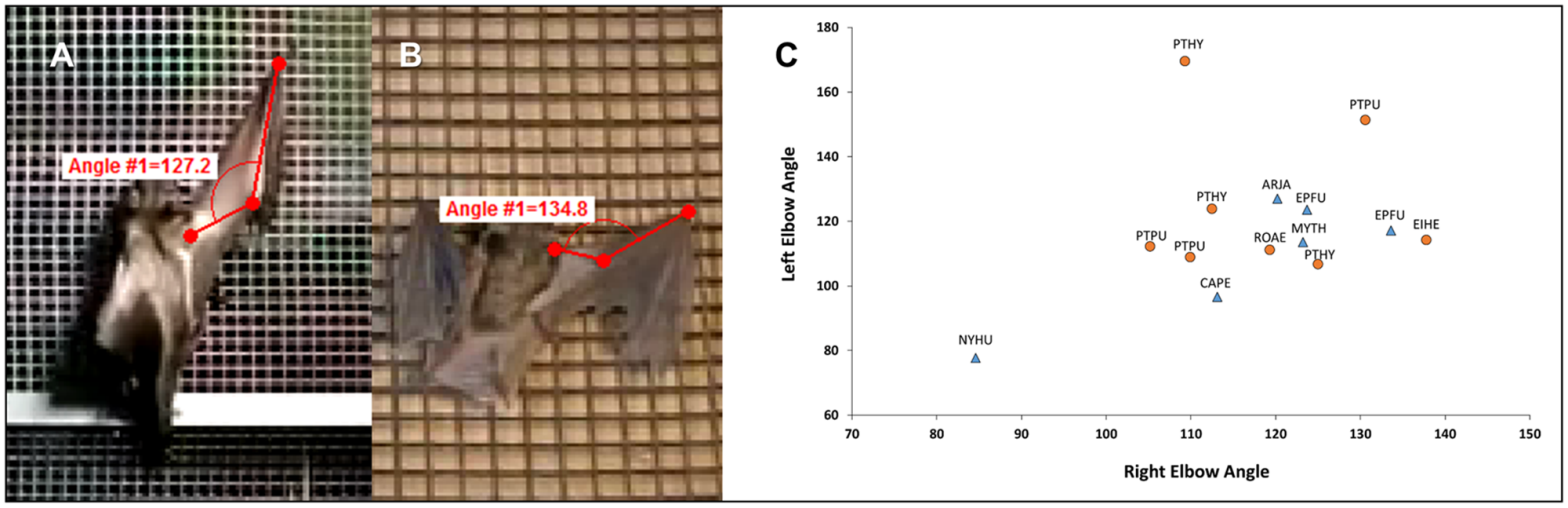
Mean angle of the elbow during climbing. A) representative megachiropteran, *Eidolon helvum* (Pteropodidae), B) representative microchiropteran, *Eptesicus fuscus* (Vespertilionidae), and C) Scatter plot of angles of right and left elbows of microchiropteran (N = 7) and megachiropteran (N = 16) bats showing complete overlap in forelimb extensions.

We measured the inside angle of the elbow joint (Fig 1A, 1B and 1C) through 1–4 left-right forearm cycles during climbing in microchiropteran and megachiropteran species. There were no significant differences between left and right elbow mean angles within microchiropterans (T = 2.78, P = 0.61), nor any significant difference between limbs within megachiroptera (T = 2.36, P = 0.52), with the exception of the two *Cynopterus brachyotis* that were subsequently excluded from the analysis. Neither of these individuals could fly so perhaps were suffering from atrophy in the front limb muscles. For the other species, data were pooled within groups and compared between megachiropterans and microchiropterans for which no significant differences were found (T = 2.26, P = 0.19); and there was nearly complete overlap in elbow angles (Fig 1C).

### Angle of the humerus to the shoulder plane during vertical climbing

**Fig 2:**
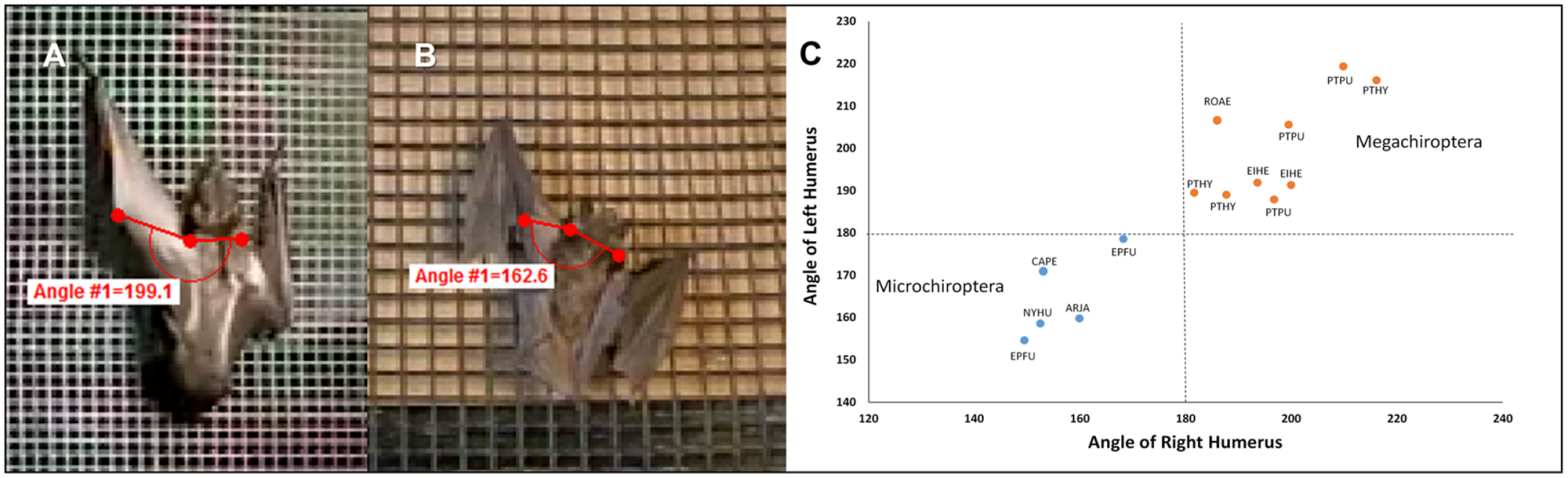
Mean angle of the humerus in relation to shoulder plane. Reference images are A) *Eidolon helvum*, B) *Eptesicus fuscus*, and C) Scatter plot of humerus angle in relation to shoulder plane shows distinctive patterns of shoulder rotation during climbing. Megachiropterans (N = 16) extended the humerus past the shoulder plane when climbing, whereas microchiropterans (N = 7) only rotated the humerus up to the shoulder plane when climbing. Microchiropterans swung their humerus during climbing ≤ 180°, whereas megachiropterans commonly swung the humerus past the shoulder plane (> 180°), thereby further extending their forelimb reach (Table 1). There were no significant differences among five species within megachiropterans (F-Ratio = 0.54, DF = 22, P = 0.66) and four species of microchiropterans (F-Ratio = 1.38, DF = 20, P = 0.30).

We measured the angle by which the humerus was swung relative to the shoulder plane by placing three landmarks, two across the top of the shoulders and one where the humerus articulates with the elbow (Fig 2A, 2B and 2C). The shoulder plane represented a set point of 180° and therefore swinging of the humerus at angles at or below the shoulder plane would be ≤ 180°, whereas swinging the humerus above the shoulder plane would be > 180°. There was no significant difference between left and right humerus movements within individual Microchiroptera (T = 2.57. P = 0.09), nor among species measured within each phylogenetic group (F-Ratio_micro_ = 1.38, DF = 20, P = 0.30), and thus these data were pooled. This was also true for both humeri in megachiropterans (T = 2.31, P = 0.61), nor between species (F-Ratio_mega_ = 0.54, DF = 22, P = 0.66), with the exception of the two *Cynopterus brachyotis* that were subsequently excluded from the analysis. Neither of these individuals could fly so perhaps had atrophy in the front limb muscles. Pooled left and right arm data were then compared between megachiropterans and microchiropterans and showed highly significant differences (T = - 8.77, P = 0.0001); and clear separation of measurements between the groups (Fig 2C).

**Table 1:** The mean angles and standard deviations (SD) of the humerus in relation to the shoulder plane in Megachiroptera and Microchiroptera during climbing. Mean angle below 180° indicates the humerus did not move past the plane of the shoulder. Mean angles above 180° indicates movement of the humerus past the shoulder plane. N = number of individuals used. There were no significant differences among species within megachiropterans (F-Ratio = 0.54, DF = 22, P = 0.66) and within microchiropterans (F-Ratio = 1.38, DF = 20, P = 0.30). The two individuals of *C*. *brachyotis* were not used in this analysis because they would barely move their forelimbs when climbing. Neither individual could fly, so perhaps they had injuries to the forelimbs. OBC did not know their history.

	*N*	Mean	SD	Deviation from 180°
Microchiropterans	7	158.1°	13.4°	-21.9°
Megachiropterans	14	198.3°	11.46°	+18.3°

### Hindlimb movements during climbing

**Fig 3:**
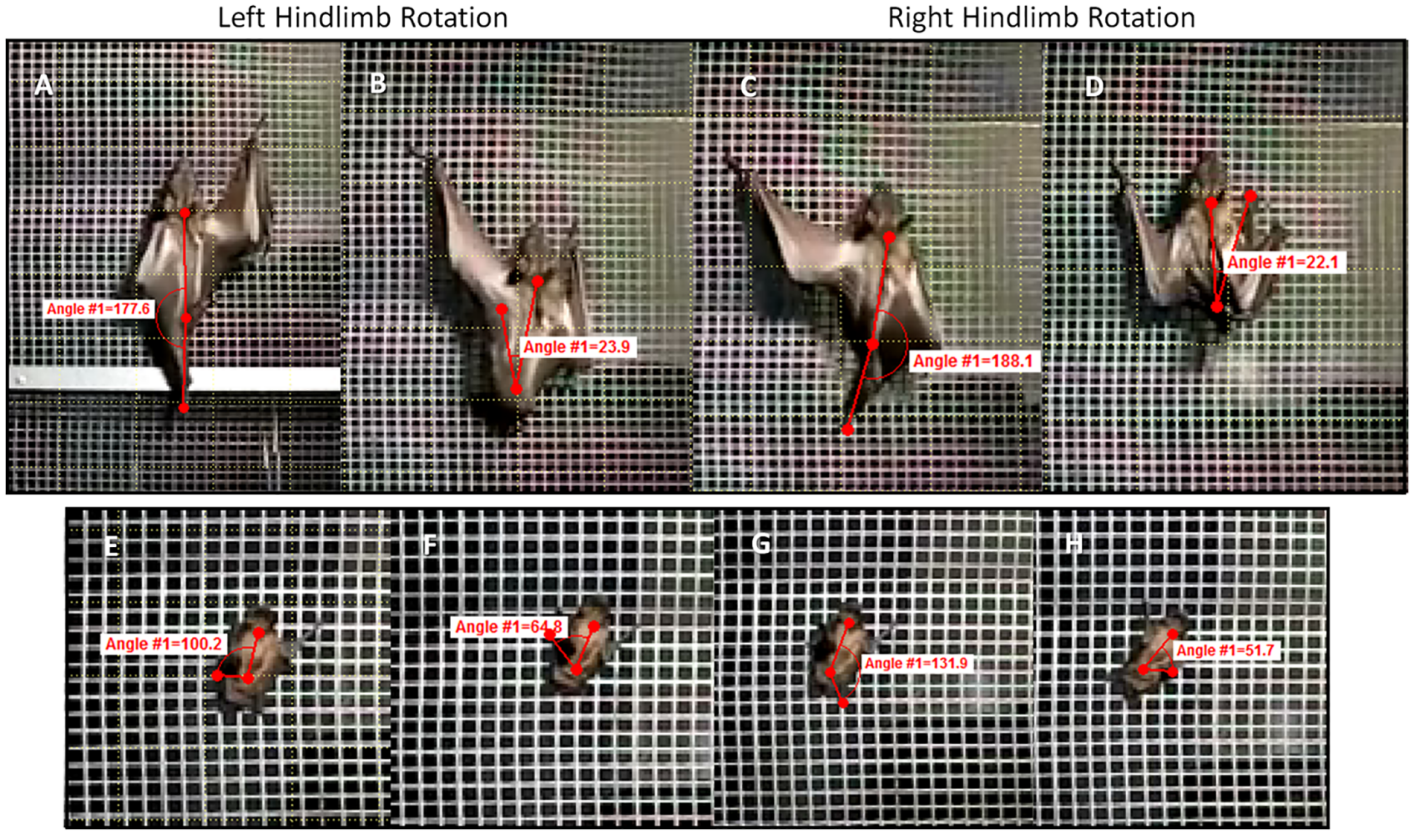
Examples of climbing hind- and forelimb mechanics. A-D) in a representative megachiropteran, *Eidolon helvum* and E-H) representative a microchiropteran, *Nycticeius humeralis*.

**Fig 4:**
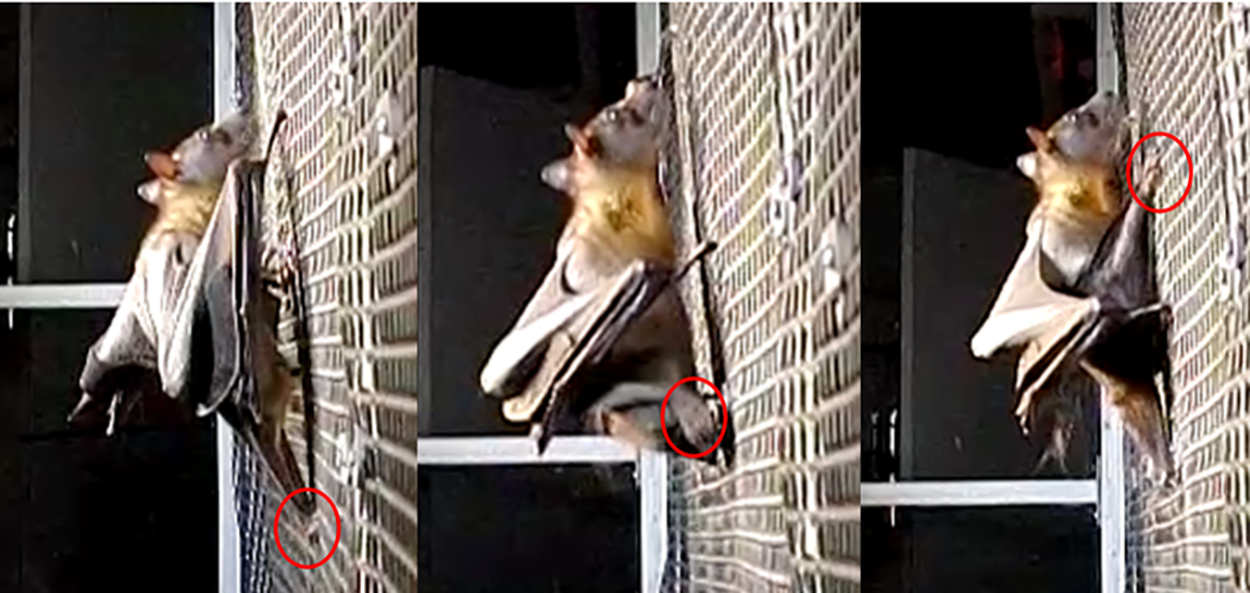
Lateral view of *Eidolon helvum* climbing. This species and other megachiropterans tested led with one of their hindlimbs to initiate climbing by swinging their foot under the forelimb and wing to position the foot near the head. As megachiropterans released their thumb claw from the screen, individuals pushed off with the hindlimb to power vertical ascent via an ipsilateral sequence gait.

When climbing vertically, megachiropterans swung their hindlimbs through a significantly greater travel arc between the fore and aft foot positions during climbing (T = 6.98, P = 0.0002) relative to the body axis than did microchiropterans (Fig 3, S1 Video). The megachiropterans *E*. *helvum*, *R*. *aegyptiacus*, *C*. *brachyotis*, *P*. *hydromelanus*, and *P*. *pumulis*, swung their hindlimbs on average of 120.2° (SD = 15.8°), whereas for the microchiropterans that would climb (*Nycticeius humeralis*, *Artibeus jamaicensis*, *Myotis thysanodes*, and *Eptesicus fuscus*), swung their hindlimbs an average of 62.6° (SD = 10.5°). *Eidolon helvum* showed the greatest hindlimb travel arc when climbing, reaching 199.3° (Table 2). In some cases in megachiropterans, protraction of the hindlimb resulted in foot placement next to, or even ahead of, the rostrum in this species (Figs 3A–3D and 4). In addition, climbing was always intiated by swinging one of the hindlimbs underneath and ahead of the forelimb, followed by reaching the forelimb on the same side of the body (ipsilateral gait) to a position ahead of the animal. Their hindlimbs appeared to be key in generating climbing thrust with the forelimbs used mostly for stability and commonly fore- and hindlimbs on the same side of the body were in motion simultaneously.

**Table 2:** Mean degrees and ranges of travel arc of the hindlimb during climbing.

SPECIES	MEAN ARC(SD)	RANGE
**Megachiroptera (Pteropodidae)**		
*Cynopterus brachotis* (N = 2)	100.1° (6.7°)	92.9°-106.1°
*Eidolon helvum* (N = 3)	140.6° (24°)	115.3°-199.3°
*Rousettus aegypticus* (N = 2)	110.2° (11.3°)	98.2°-161.4°
*Pteropus hydromelanus* (N = 2)	129.6° (2.4°)	126.3°-132.9°
*Pteropus pumilus* (N = 3)	121.9° (2.7°)	117.2°-125.7°
**Microchiroptera**		
*Artibeus jamaicensis* (N = 1)	63.2° (11.6°)	55.0°-71.4°
*Eptesicus fuscus* (N = 2)	55.2° (10.1°)	40.6°- 64.0°
*Myotis thysanodes* (N = 2)	61.5 (7.6°)	54.7–72.9
*Nycticeius humeralis* (N = 1)	68.3° (9.5°)	54.5°-77.1°

**Fig 5:**
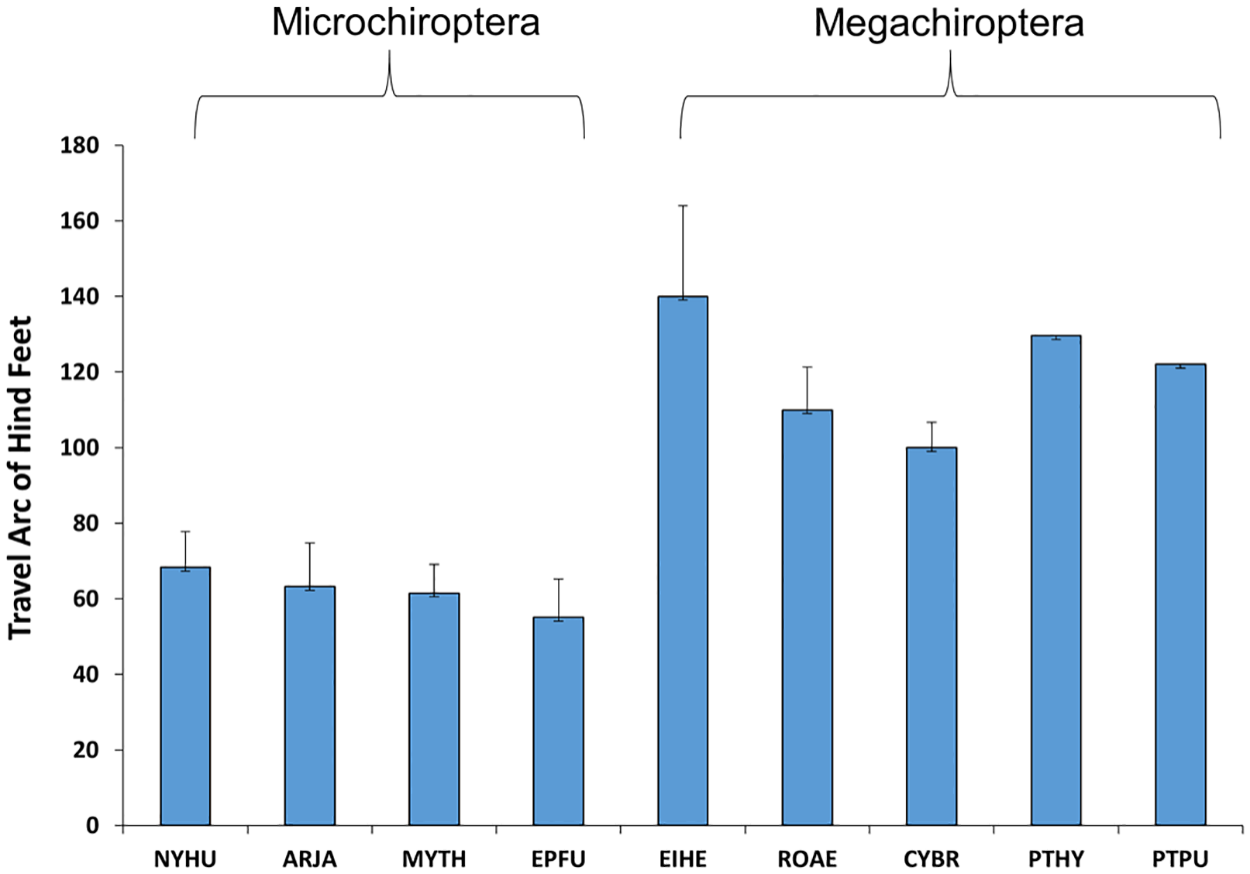
Means and standard deviations (error bars) of travel arcs of hindlimbs during protraction in four microchiropterans. (NYHU = *Nycticeius humeralis*, N = 1; ARJA = *Artibeus jamaicensis*, N = 1; MYTH = *Myotis thysanodes*, N = 2; and EPFU = *Eptesicus fuscus*, N = 2; versus five megachiropterans (EIHE = *Eidolon helvum*, N = 3; ROAE = *Rousettus aegyptiacus*, N = 2; CYBR = *Cynopterus brachyotis*, N = 2; PTHY = *Pteropus hypomelanus*, N = 6; and PTPU = *Pteropus pumilus*, N = 3; during climbing.

In microchiropterans, swinging extent of the hindlimbs during climbing was typically at oblique angles to the body plane and never passed ahead of the forelimbs (Fig 3E–3H, S2 Video). Although one could argue that the tail membrane (uropatagium) may have restricted the ability to protract the hindlimbs during climbing, the Jamaican fruit bat (*A*. *jamaicensis*), which has a greatly reduced uropatagium, showed the same mechanics as the other microchiropterans (Fig 5, Table 2). In microchiropterans, movement of limbs was always done using a contralateral sequence gait (i.e. LF, RH, RF, LH) with three limbs in contact with the surface as the fourth limb was moved. There was no significant differences among microchiropteran species (H = 4.66, P = 0.19) or within microchiropterans (H = 15.1, P = 0.004) in travel arcs of the hindlimbs. However, there was a significant difference between megachiropterans versus microchiropterans (T = -6.98, P = 0.0002).

### Behaviors associated with horizontal walking

When placed on a horizontal surface, microchiropterans either immediately attempted to take flight or walked or scurried along the platform using diagonally alternating, contralateral, limb motions. As in climbing, one of the forelimbs was used to initiate locomotion by reaching forward and followed by a contralateral hindlimb with the hindfoot being used to push the animal forward. The thumb claws were held perpendicular to the movement of the forelimb and thus did not appear to be used to grip and pull the bat forward. Maximum swing of the hindlimb in *N*. *humeralis* was 58.7° when walking which was comparable to its use in climbing. Our results did not differ from those of Lawrence [3] with all tested microchiropteran species using the same footfall patterns and diagonal sequence gaits. Thus, there was no discernible variation between climbing and walking in terms of general mechanics.

We compared humerus swing angles relative to the shoulder plane of *N*. *humeralis* when climbing versus walking. First, we tested for significant differences between right and left arms and found no significant differences (climbing: T = -1.68, P = 0.28; walking: T = 1.44, P = 0.19). Thus, left and right arm data were pooled and compared between walking and climbing. Mean swing angle of the humerus relative to the shoulder plane when climbing was 209.8° (SD = 26.8°, N = 8), whereas for walking was 234.2° (SD = 40.3°, N = 8). As with climbing, the humerus never extended past the shoulder plane in *N*. *humeralis* when walking and although *N*. *humeralis* showed less protraction of the humerus relative to the shoulder plane when walking than when climbing, this difference was not significant (T = -1.42, P = 0.17). We also compared swing arc of the hindlimbs in *N*. *humeralis* while walking and climbing and there were no significant differences (T = - 0.34, P = 0.74; mean climbing = 71.5°, SD = 18.3°; mean walking = 73.8°, SD = 7.3°). The outcome was similar for *E*. *fuscus* for the hindlimb angle (T = - 0.01, P = 0.99, mean walking = 76.9°, mean climbing = 76.9°) and for humerus angle (T = - 0.81, P = 0.45, mean walking = 140.4°, mean climbing = 132.1°) as they were for *M*. *thysanodes* (hindlimb swing angle T = - 0.02, P = 0.31, mean walking 61.5°, mean climbing 77.2°) and for humerus angle (T = - 1.32, P = 0.21, mean walking 169.9°, mean climbing 171.8°). The Jamaican fruit bat (*A*. *jamaicensis*) would not walk and thus we provide no comparative data on this species.

**Fig 6:**
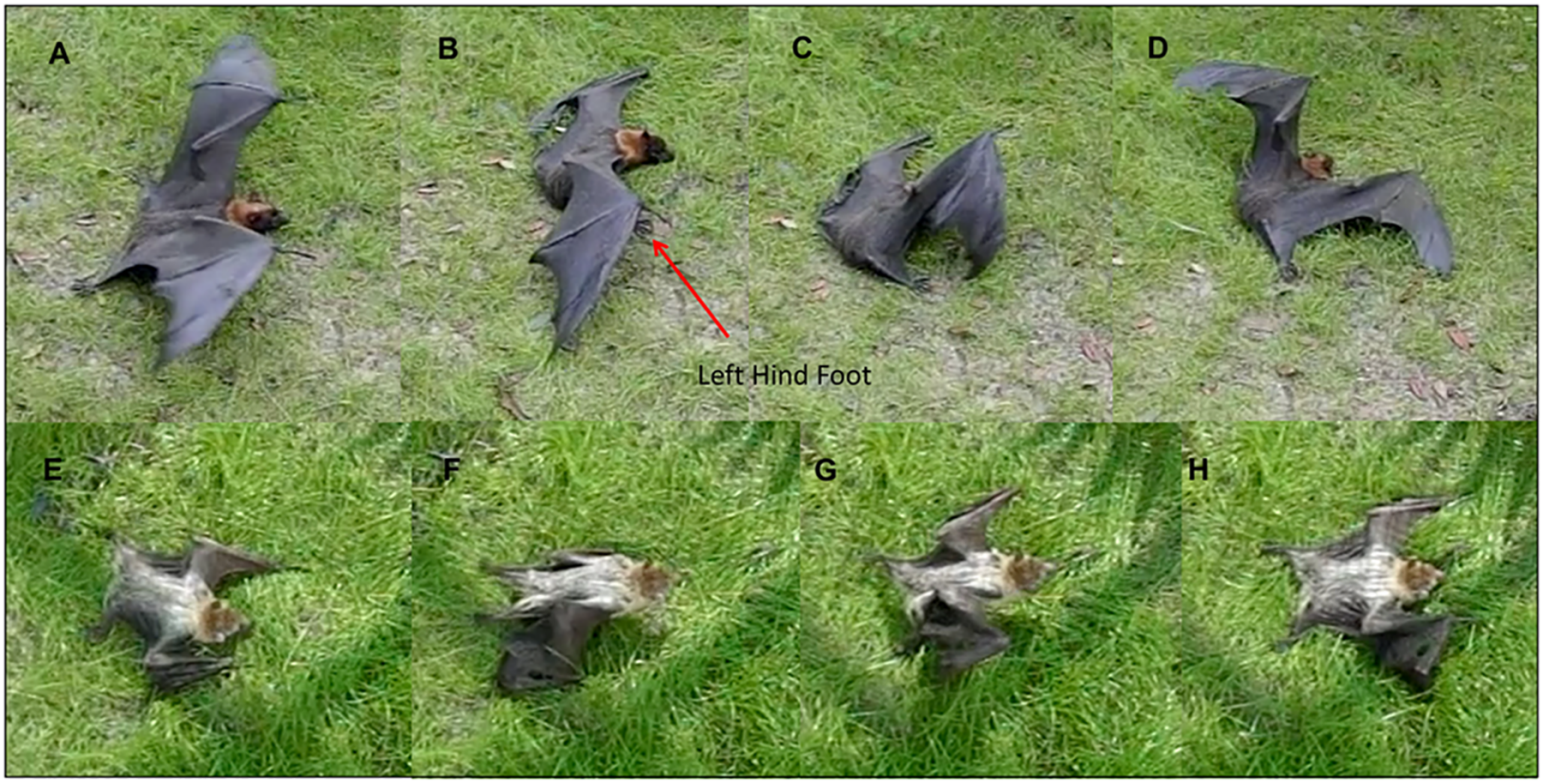
Ground locomotion in megachiropterans. (A-D) *Pteropus hypomelanus* (N = 2) attempting to walk on the ground using hindlimb propulsion and lifting the forelimbs off the ground resulting in an awkward belly-flopping movement. (E-H) *Pteropus pumilus* (N = 2) showing pulling behavior wherein the hindlimbs are not used but the thumb claws are employed to pull the individual's body temporarily off the ground and foreword.

Both *C*. *brachyotis* and *R*. *aegyptiacus* immediately initiated attempted to launch into flight, rather than walk, so we provide no walking data for them. However, both *E*. *helvum* and *P*. *hypomelanus* initiated terrestrial locomotion when placed on the ground and swung their hindlimbs under and sometimes ahead of their forelimbs to positions that mimicked their climbing locomotion (Fig 6A–6D, example of ground walking in *P*. *hypomelanus*, S3 Video). In addition, individuals of *Pteropus pumilus* simply pulled themselves along the ground using only the forelimbs and thumb claws and pushing off into a bounding type of locomotion (Fig 6E–6H, S4 Video). For megachiropterans in general, terrestrial locomotion when the hindlimbs were employed appeared awkward and nonlinear. Such nonlinear locomotion can be energetically expensive [9].

### Comparison of climbing and walking gaits

**Fig 7:**
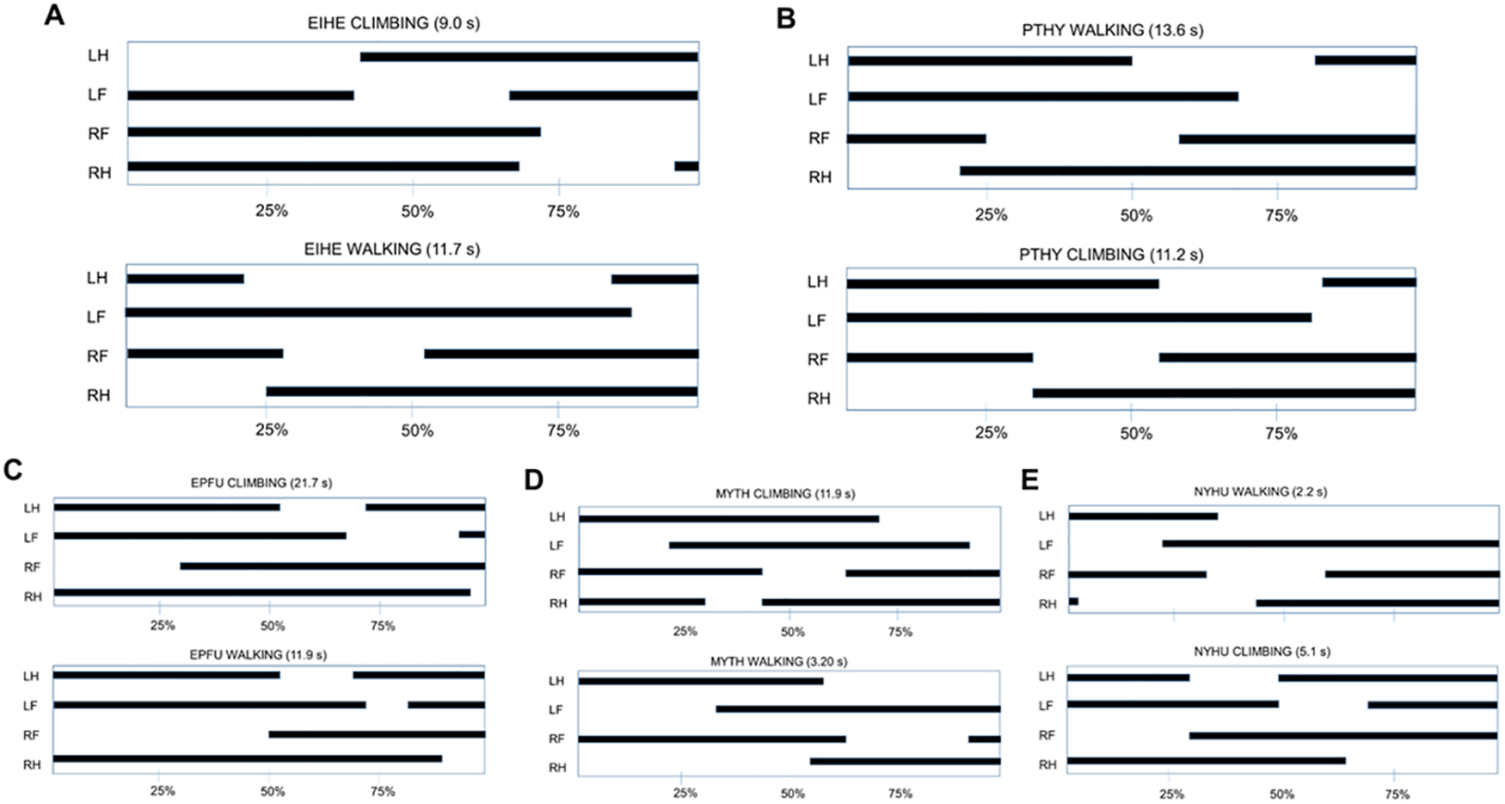
Gait graphs constructed following the methodology of Hildebrand (1966) show limb motions at initiation of climbing and walking in representative individuals. (A-B) megachiropterans *E*. *helvum* (EIHE) and *Pteropus hypomelanus* (PTHY); (C-E) microchiropterans *E*. *fuscus* (EPFU) *Myotis thysanodes* (MYTH) and *N*. *humeralis* (NYHU). Black lines indicate when the foot is in contact with the vertical or horizontal surface and gaps indicate elevated movement of the limbs. Footfall patterns of limb motion in megachiropterans were consistently initiated by one of the hindlimbs followed by the forelimbs on the same side of the body (ipsilateral). This was true for climbing and walking. In microchiropterans, footfall patterns were consistently initiated by one of the forelimbs followed by a diagonally located hindlimb (contralateral) for both walking and climbing.

Comparing initiation sequences of walking and climbing using Hildebrand diagrams [10] in two representative megachiropterans [*E*. *helvum* (EIHE) and *Pteropus hypomelanus* (PTHY)] and three representative microchiropteran species [*E*. *fuscus* (EPFU), *M*. *thysanodes* (MYTH), and *N*. *humeralis)*] showed that megachiropterans (Fig 7A and 7B) initiated climbing and walking by protraction of one of the hindlimbs and used ipsilateral gaits in both walking and climbing, whereas microchiropterans (Fig 7C, 7D and 7E) initiated climbing and walking by protraction of one of the forelimbs and used contralateral gaits in both walking and climbing.

### Performance in climbing and walking

Predicatively, steep slopes should cause larger animals to slow more severely than smaller animals [11, 12] and in vertical climbing, the effects of gravity should cause slower climbing in larger bodied individuals all else being equal [13, 14, 15, 16]. Thus, we tested the hypothesis that *N*. *humeralis* (mass approximately 10g, [17]) should accelerate from a dead start through two cycles (i.e. each of the four limbs being elevated and lowered back to the ground) at a faster rate than *E*. *helvum* (approximately 230g, [18]). In order to scale the distance moved relative to body size over time, we used head length (HL) of each species as measured using digital calipers on the computer screen. The microchiropteran *N*. *humeralis* moved at a pace of 1.5 HL/s when walking, whereas the megachiropteran *E*. *helvum* moved at a pace of 2.2 HL/s, approximately 32% faster when climbing.

**Fig 8:**
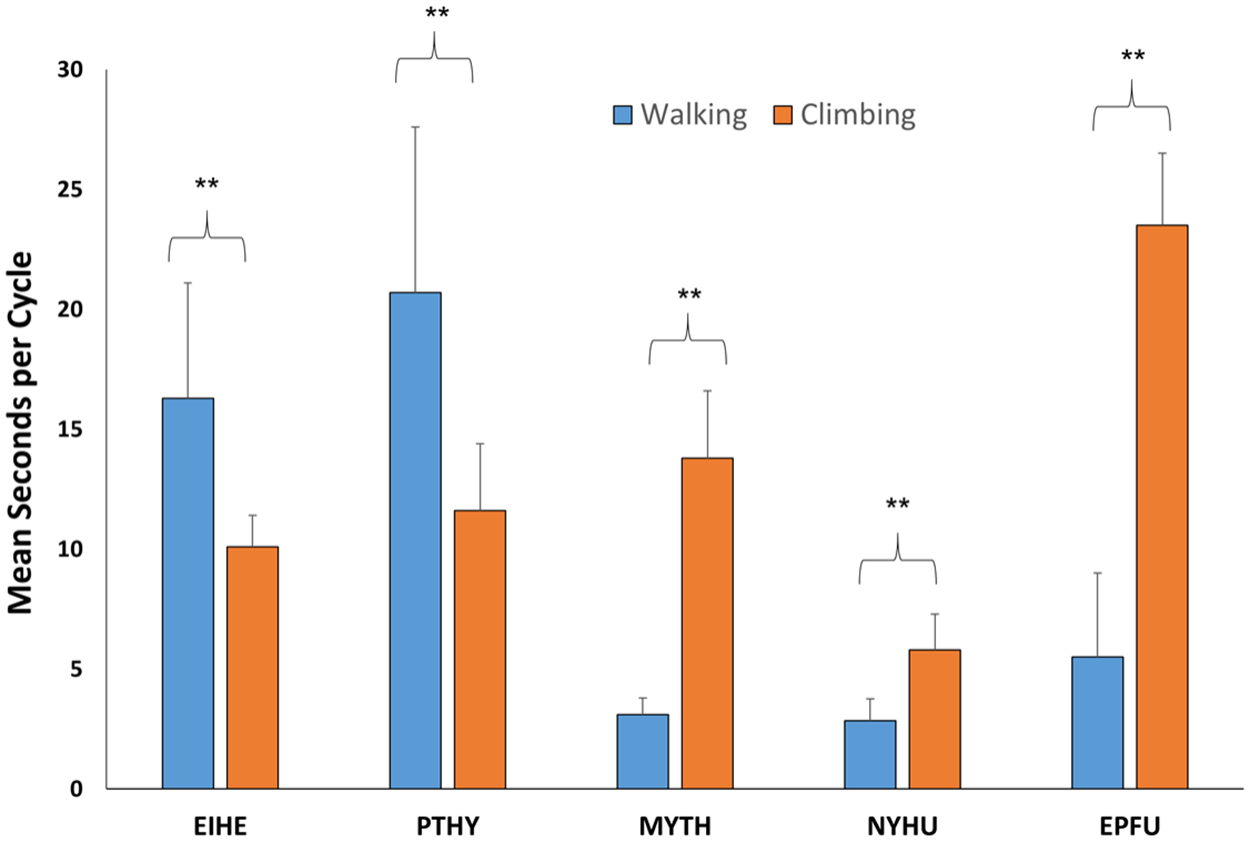
Bar graph of mean seconds per cycle (one cycle equaled all four limbs lifted and replaced) for walking and climbing. In the megachiropteran, *Eidolon helvum*, mean of seven walking cycles of three individuals = 16.3s (SD = 4.8), mean of seven climbing cycles of four individuals = 10.1s (SD = 1.34s), Z-score = -3.23, P < 0.005 and in the megachiropteran *Pteropus hydromelanus*, mean of four walking cycles of two individuals = 20.7s (SD = 2.3s), mean of four climbing cycle of two individuals = 11.6s (SD = 2.9s), Z-score = -3.23, P < 0.05). In microchiropterans the opposite was found. In *Myotis thysanodes*, mean of five walking cycles of two individuals = 3.0s (SD = 0.7s), mean of five climbing cycles of two individuals = 13.8s (SD = 2.8s), Z-score = -2.65, P < 0.05, in *Nycticeius humeralis*, mean of five walking cycles of one individual = 2.8s (SD = 0.9s), mean of five climbing cycles of one individual = 5.8s (SD = 1.5s), Z-score = -2.60, P < 0.05, and *Eptesicus fuscus*, mean of two walking cycles of one individual = 11.2s (SD = 0.98s), mean of two climbing cycles of one individual = 23.5s (SD = 0.7s), Z-score = -2.60, P < 0.05].

In addition, we tested if there were significant differences in mean limb cycle times between walking and climbing in microchiropteran and megachiropteran species that would both walk and climb. Unfortunately, *C*. *brachyotis* and *R*. *aegyptiacus* would immediately launch into flight when released on a horizontal platform or, in the case of *P*. *pumilus*, would only pull themselves along the ground using the forelimbs and thus walking could not be measured in these species. Immediate launching into flight was also true of *C*. *perspicillata* and *A*. *jamaicensis*, and thus we could not compare limb cycling rates in these two microchiropteran species. Both microchiropteran and megachiropteran species we could compare, they showed significant differences in cycling times when walking versus climbing, but the differences were opposite for each group. The megachiropterans cycled their limbs significantly faster when climbing as compared to walking, [*Eidolon helvum*, mean of seven walking cycles of three individuals = 16.3s (SD = 4.8), mean of seven climbing cycles of three individuals = 10.1s (SD = 1.34s), Z-score = -3.23, P < 0.005; *Pteropus hydromelanus*, mean of four walking cycles of two individuals = 20.7s (SD = 2.3s), mean of four climbing cycle of two individuals = 11.6s (SD = 2.9s), Z-score = -3.23, P < 0.05), whereas in microchiropterans the opposite was true (*Myotis thysanodes*, mean of five walking cycles of two individuals = 3.0s (SD = 0.7s), mean of five climbing cycles of two individuals = 13.8s (SD = 2.8s), Z-score = 2.65, P < 0.05; *Nycticeius humeralis*, mean of five walking cycles of one individual = 2.8s (SD = 0.9s), mean of five climbing cycles of one individual = 5.8s (SD = 1.5s), Z-score = -2.60, P < 0.05, and *Eptesicus fuscus*, mean of two walking cycles of one individual = 11.2s (SD = 0.98s, S5 Video), mean of two climbing cycles of one individual = 23.5s (SD = 0.7s), Z-score = -2.60, P < 0.05] (Fig 8).

## Discussion

We found that the fundamental aspects of behavior, mechanics, and kinematics of climbing and walking were significantly different between megachiropteran and microchiropteran bats. In essence, megachiropterans appear to have evolved suites of integrated behaviors, adaptive characters, and kinematic dimensions for arboreality [19, 20, 21]. These adaptations are functionally independent from the evolution of flight and not observed in microchiropterans. In addition, megachiropterans appeared to have lost, or at least rarely used, much of the basic mechanics associated with terrestrial locomotion, another concordant pattern observed in specialized arboreal states [22, 23]. In addition, microchiropterans showed traits and behaviors commonly observed in terrestrial mammals when placed in an arboreal environment. These included slow, cautious movements, decreased stride frequency, the use of a contralateral gait for stability, and retained three points of contact with the substrate [24, 25]. The contralateral gait used by microchiropterans when climbing was the same gait they used when walking and represents mechanics and kinematics that has changed little from the earliest evolved tetrapods [26, 27].

The morphological differences between megachiropterans and microchiropterans is profound [28, 29] and our analysis of basic nonflight locomotion indicates that these groups are significantly divergent in how they conform to being placed in arboreal vesus terrestrial environments. Microchiropterans showed a range of terrestrial abilities, but the fundamental platform for walking, and climbing, was a basic tetrapodal gait (using contralateral limb movements and moving a single limb at a time with three points of contact). Megachiropterans functioned uniquely similarly to arboreal mammals in climbing and showed similar unease when placed on the ground that other arboreally adapted mammals show [23, 30].

One would envision that in the evolution of a monyphyletic group, individuals that share similar environments and selective pressures would derive similar morphologies and behaviors. However, frugivorous microchiropterans that operate in a very similar ecomorphological space as do megachiropterans lack similar character states or behaviors consistent with arboreality. Microchiropterans exhibit behaviors similar to those observed when terrestrial mammals are placed in an arboreal situation [11]. For example, the Jamaican fruit bat (*A*. *jamaicensis*) is a New World, tree-roosting species that forages on large fruits, very similar to what we observe in nonfruigivorous species of megachiropterans. The selective pressures that adapted bat species to being frugivorous, did not result in the same nonflight locomotor mechanics for climbing and walking in microchiropterans and megachiropterans. As a curious aside, the white-winged vampire bat, *Diamus youngi*, which preys on night-roosting birds [31], is commonly cited to be one of the most arboreally adept microchiropterans. Yet, limited footage of these bats climbing head-first shows them using a contralateral gait like the microchiropterans tested in our study (https://www.youtube.com/watch?v=LTxxT4SZu4k). Thus, even the most arboreal of microchiropteran species, as of yet, have not show the more agile climbing mechanics of megachiropterans.

That megachiropterans did not revert to a basic tetrapod locomotor gait when placed on the ground was intriguing and might reflect an evolutionary tradeoff for efficient arboreal locomotion. Similarly, two-toed sloths (*Choloepus hoffmanni*) when on the ground locomote by slowly dragging their bodies forward [23]. The megachiropterans we tested struggled to maintain linearity when attempting walking mechanics. Their long thumbs and claws, although adaptive for climbing, appeared to be detrimental in terrestrial locomotion and individuals typically held their forelimbs off the ground while abducting their hindlimbs under and ahead of their body, the latter being similar to the climbing mechanics we observed. This led to a swinging back and forth of the bats body while moving forward resulting in nonlinear progress. Halsey [32] showed that when an animal's direction of travel is not linear, its energy costs are intensified 10–15 times. Perhaps this is the reason why some individuals simply pulled themselves along the ground using their thumb claws as this resulted in more linearity from point A to B. The comparative energetic costs of these terrestrial locomotor modes versus climbing would indeed be an interesting line of inquiry. Data from wild chimpanzees show that they expend 10 times more energy per day on terrestrial versus vertical travel [33]. In addition, arboreal specialist primates called sifakas (*Propithecus verreauxi*) use a form of galloping and leaping that mimicked their highly derived arboreal mechanics [34]. The microchiropterans we tested, and tested in other published studies, walk or scramble with mechanics that can be readily recognized as basic tetrapod terrestrial locomotion (described above).

The geologic record suggests that megachiropterans evolved between 20 and 25 million years later in the fossil record than microchiropterans, with no known transitional stages [1]. The question becomes, if megachiropterans are an evolutionary offshoot of microchiropterans, why do they possess character states and locomotor skills beyond the arboreal capacity of microchiropterans and why have they apparently lost many of the basics of terrestrial locomotion? We propose the hypothesis that these groups of bats are derived from different baseline mechanical platforms. How this happened remains an open question.

Thinking more broadly, the arboreal nature of megachiropterans may give insight into why they are so restricted ecomorphologically as compared to microchiropterans? If the immediate ancestor to megachiropterans went through intense selection for arboreal characters, such selective pressures for specialization may have inherently constrained future diversity in descendant lineages. The lack of ecomorphological diversity in megachiropterans, as compared to microchiropterans, currently has no satisfactory answer.

There are two ways in which evolutionary constraint may be manifested: lack of genetic diversity (variation) to allow for phenotypic expression and thus positive selection, or a lack of ecological diversity to which populations are exposed [35]. The latter does not appear to be the case as megachiropterans occur in areas, in the geologic past and present, that correspond with the highest ecomorphological diversity of microchiropterans [36]. However, we should be careful in that the fossil record of bats is woefully incomplete.

The lack of laryngeal echolocation ability in megachiropterans also remains inexplicable. If megachiropterans were derived from a microchiropteran ancestor, why the loss of echolocation for greater visual acuity? Curiously, there are no known arboreally specialized mammals, including tree-shrews (Scandentia, Tupaiidae), that emit sonar or echolocation. However, it is well known that several species of extant terrestrial shrews use sonar and echolocation to navigate in darkness [37, 38, 39, 40]. Therefore, hypothetically, microchiropterans may have inherited, along with basic terrestrial nonflight locomotion, a primitive echolocation system from a terrestrial shrew-like ancestor [41]. Separating megachiropterans from this hypothetical origin of microchiropterans provides for an interesting resolution as to why megachiropterans lack laryngeal echolocation.

Our study raises more questions than we can answer thus far and will likely lead to some contentious responses from specialists. However, we predict that the nonflight locomotor patterns we underline in this paper will be consistent within and across megachiropterans and microchiropterans when more species are tested in more detail. The mechanics and kinematics described herein appear fundamentally ingrained in each group and to represent the deep history of bats before the evolution of flight occurred. More work clearly needs to be conducted on nonflight locomotor mechanics in bats, and we believe such inquiry will help uncover the illusive evolutionary origin of flight in mammals.

## Supporting information

S1 Video Megachiropteran *Eidolon helvum* (Pteropodidae) free-climbing. Notice initiation of climb using rear-limb, ability to extend the humerus past the should plane, ability to abduct and protract the hindlimbs placing the hind foot near the head, and ipsilateral gait with only 1–2 points of contact. Filmed at 300 Hz.
(AVI)

S2 Video Microchiropteran, *Myotis thysanodes* (Vespertilionidae), free-climbing. Note initiation of climb using forelimb, relatively limited extension of the humerus to positions below the shoulder plane, relatively limited abduction and protraction of the hindlimbs, and the use of a basic contralateral gait with 3 points of contact. Filmed at 300 Hz.
(MP4)

S3 Video Megachiropteran *Pteropus hypomelanus* (Pteropodidae) moving on the ground. Note ipsilateral movement of limbs with most propulsion coming from the hindlimbs. This individual also appears to be lifting its elongated thumb claws to avoid contact with the ground. Filmed at 300 Hz.
(WMV)

S4 Video Megachiropteran *Pteropus pumilus* (Pteropodidae)using its thumb claws to pull itself along the ground. Note the lack of hindlimb employment. Filmed at 300 Hz.
(WMV)

S5 Video Microchiropteran *Eptesicus fuscus* (Vespertilionidae) walking on a platform. Note the contralateral gait which affords stability at slow speeds as observed in basic tetrapods. Filmed at 300 Hz.
(WMV)
